# Medical Society of Pennsylvania

**Published:** 1881-02-20

**Authors:** 


### *MEDICAL SOCIETY OF PENNSYLVANIA.*

Transactions of the Medical Society of the State of Pennsylvania at its 13th Annual Session held at Altoony, May, 1880. Vol. XIII, Part 1. Published by the Society, Philadelphia, 1880. Collins Printer, 205 Jayne Street.

This is a large and creditable volume of 494 oc. pages, containing elaborate and valuable papers on many important subjects. It includes reports from both the Central and Local or County Societies.

We have not space for a full notice of the articles from the several authors. Those read or delivered before the Society proper, are as follows:

President's Address, by Dr. Nebinger; Report on Medical Legislation, by Dr. R L. Sebbit; Fibroid Tumors of the Womb, by Dr. Wm. Goodell; History of Obstetrics in Pennsylvania, by Dr. J. T. Carpenter; New Remedies in the Treatment of Skin Diseases, by Dr. John V. Shoemaker; Address on Surgery, by Dr. John H. Packard; Hyper Distension of the Air Cells as a Therapeutic Measure, by Dr. J. S. Cohen; Treatment of Asthma, by Dr. Wm. Pepper; Address on Hygeine, by Dr. Benj. Lee; An Open Adjustable Appliance for the Treatment of Spinal Curvature, by Dr. E. H. Coover; Bromide of Ethyl as an Anæsthetic in Practical Surgery, by Dr. John B. Roberts; Note on the Alkaloids of Cinchona, by Dr. Benj. Lee; Enumeration, Classification and Causation of Idiocy, by Dr. Isaac N. Kerlin; Report on an Operation for the Removal of an Unusually Large Submucous Fibroid of the Uterus, by Dr. S. T. Davis; The Normal Axis of the Sole of the Human Foot, by Dr. Benj. Lee; Puerperal Septicæmia, by Dr. Wm. H. Parish.

